# Epigallocatechin Gallate-Primed Bone Marrow Mesenchymal Stem Cell-Derived Extracellular Vesicles Attenuate Endothelial Senescence by Modulating PI3K/AKT/mTOR Signaling and PINK1-Associated Mitochondrial Quality Control

**DOI:** 10.3390/life16101737

**Published:** 2026-10-16

**Authors:** Yifan Tao, Yangli He, Tian Xie, Min Zeng

**Affiliations:** Institute of Cardiovascular Diseases, Hainan Medical University, Haikou 571199, China; taoyf009@126.com (Y.T.);

**Keywords:** epigallocatechin gallate, extracellular vesicles, endothelial senescence, mitochondrial quality control, PINK1/Parkin, PI3K/AKT/mTOR signaling

## Abstract

(1) Background: Oxidative stress-induced endothelial senescence is associated with mitochondrial dysfunction, impaired mitochondrial quality control, and pro-inflammatory signaling. We examined whether extracellular vesicles (EVs) derived from epigallocatechin gallate (EGCG)-primed bone marrow mesenchymal stem cells protect senescent human aortic endothelial cells (HAECs). (2) Methods: HAEC senescence was induced with H_2_O_2_. EVs from untreated and EGCG-primed BMSCs were isolated and characterized. Proliferation, senescence, apoptosis-associated proteins, mitochondrial morphology and membrane potential, mitophagy-related markers, senescent-HAEC-directed RAW264.7 macrophage migration, and PI3K/AKT/mTOR signaling were evaluated using CCK-8 and EdU assays, senescence-associated β-galactosidase staining, Transwell migration assays, JC-1 staining, transmission electron microscopy, mitochondria–lysosome colocalization, Western blotting, Mdivi-1, PINK1 knockdown, and the PI3K activator 740 Y-P. (3) Results: EGCG-primed BMSC-derived EVs were taken up by HAECs and alleviated H_2_O_2_-induced senescence, increasing proliferation. And reducing β-Gal+ cells from 36.47% to 10.17%, and RAW264.7 migration from 720.33 to 123.67. They restored mitochondrial membrane potential, enhanced PINK1/Parkin-associated mitochondrial quality control, and suppressed PI3K/AKT/mTOR phosphorylation. Mdivi-1, PINK1 knockdown, and 740 Y-P attenuated these protective effects relative to the corresponding H_2_O_2_ + intervention controls. (4) Conclusions: EGCG priming enhanced the protective activity of BMSC-derived EVs against H_2_O_2_-induced endothelial senescence, with the involvement of PI3K/AKT/mTOR inhibition and PINK1-associated mitochondrial quality control.

## 1. Introduction

Vascular endothelial cell senescence is increasingly recognized as an active contributor to endothelial dysfunction and atherogenesis. Recent experimental evidence integrating human plaque analyses, Apoe-deficient mice, and endothelial cell models showed that atheroprone oscillatory shear stress promotes endothelial senescence and plaque formation through mitochondrial DNA-cGAS-STING signaling [1]. More broadly, senescence across vascular cell types contributes to vascular inflammation and lesion progression [1,2]. Atherosclerosis (AS) is the pathological basis of major cardiovascular diseases, including myocardial infarction, stroke, and peripheral arterial disease. Under oxidative, inflammatory, and metabolic stress, human aortic endothelial cells (HAECs) can undergo premature senescence, accompanied by impaired proliferation, migration, and angiogenic capacity and by increased release of senescence-associated secretory phenotype (SASP) factors, including IL-6, TNF-α, and iNOS [3]. These changes compromise endothelial barrier function and promote inflammatory cell infiltration and lipid deposition, thereby contributing to plaque formation and instability [4]. Limiting endothelial senescence and restoring endothelial homeostasis may therefore help address vascular aging and AS-associated endothelial dysfunction.

Mitochondrial homeostasis is essential for normal endothelial cell function. Mitochondrial damage and excessive reactive oxygen species accumulation are prominent features of senescent endothelial cells [5]. Mitophagy, the selective autophagic removal of damaged or dysfunctional mitochondria, contributes to mitochondrial quality control, energy metabolism, and cellular stress resistance [6]. Impaired mitophagy allows dysfunctional mitochondria to accumulate and sustains oxidative and inflammatory stress, which may further aggravate endothelial senescence and AS progression [7].

Extracellular vesicles (EVs) derived from bone marrow mesenchymal stem cells (BMSCs; MSCs-EVs) are nanoscale extracellular vesicles with low immunogenicity, biocompatibility, and cargo delivery capacity [8,9]. By transferring proteins, microRNAs, and bioactive lipids to recipient cells, MSCs-EVs can influence proliferation, apoptosis, autophagy, and angiogenesis [10]. Pharmacological priming has been investigated as a strategy to alter the biological activity of stem-cell-derived EVs. Epigallocatechin gallate (EGCG), a major bioactive component of green tea, has antioxidant, anti-inflammatory, and anti-senescent properties [11] and can regulate autophagy and mitochondrial function [12]. EVs-based delivery of EGCG has also been explored [13]. However, whether EVs from EGCG-primed BMSCs (MSCs^EGCG^-EVs) protect endothelial cells from oxidative stress-induced senescence through mitochondrial quality control remains unclear.

The PINK1/Parkin pathway is a canonical regulator of mitophagy and inflammation [14], whereas PI3K/AKT/mTOR signaling is a major regulator of cell growth, autophagy, and inflammation [15,16]. In this study, we isolated and characterized MSCs-EVs and MSCs^EGCG^-EVs and established an H_2_O_2_-induced HAEC senescence model. We evaluated their effects on endothelial senescence and examined the involvement of PINK1-associated mitochondrial quality control and PI3K/AKT/mTOR signaling using pharmacological modulation and PINK1 knockdown.

## 2. Materials and Methods

### 2.1. Animal Procedures

Male Sprague-Dawley rats were obtained from Sipeifu (Beijing) Biotechnology Co., Ltd. (Beijing, China). All animal procedures were approved by the Institutional Animal Care and Use Committee of Hainan Medical University (approval No. SY: 2026-03-006) and reported in accordance with the ARRIVE guidelines.

Four-week-old rats were housed in a specific-pathogen-free facility at 22 °C with 50–60% relative humidity under a 12 h light/dark cycle, with three rats per cage. Autoclaved corncob bedding and environmental enrichment items were replaced weekly. Standard laboratory chow and sterile water were available ad libitum.

Before bone collection, rats received meloxicam (1–2 mg/kg, subcutaneously) to minimize pain and distress. Euthanasia was performed using a gradual-fill CO_2_ method (20–30% of chamber volume per minute), followed by confirmation of death. The femurs and tibias were then dissected aseptically, cleared of adherent soft tissue, and used immediately for BMSC isolation. Body weight, tibia length, and successful BMSC isolation were recorded.

### 2.2. Cell Culture and Senescence Induction

Rat BMSCs were isolated from the femurs and tibias of 4-week-old male Sprague-Dawley rats. Under sterile conditions, the bone marrow cavity was flushed with DMEM (Hyclone, Logan, UT, USA) using a 1 mL syringe. The resulting cell suspension was centrifuged at 300× *g* for 5 min, and the pellet was resuspended in DMEM supplemented with 10% fetal bovine serum (FBS; Hyclone, USA) and 1% penicillin/streptomycin. The cells were incubated at 37 °C in a humidified atmosphere containing 5% CO_2_. Non-adherent cells were removed after 48 h, and adherent BMSCs were cultured to 80% confluence.

HAECs (human aortic endothelial cells) were purchased from ScienCell Research Laboratories (Carlsbad, CA, USA). HAECs were maintained in complete endothelial cell culture medium (Servicebio, Wuhan, China) containing 5% FBS [17]. To induce senescence, passages 3–6 HAECs were treated with 50 μmol/L of H_2_O_2_ for 2 h and then cultured in fresh medium for 48 h, thereby inducing the senescent phenotype.

### 2.3. Multilineage Differentiation of BMSCs

Third-passage BMSCs were cultured in adipogenic, osteogenic, or chondrogenic induction medium (Sigma, Roedermark, Germany) for 21 days. Differentiation was evaluated using Oil Red O, Alizarin Red S, and Alcian Blue staining (Solarbio, Beijing, China), respectively.

### 2.4. EVs Isolation and Characterization

When BMSCs reached 80% confluence, the culture medium was replaced with serum-free medium. Conditioned medium was collected after 48 h from BMSCs at passages 3–6 and subjected to sequential differential centrifugation. The medium was centrifuged at 200× *g* for 10 min to remove cells and at 2000× *g* for 10 min to remove cellular debris. The supernatant was then centrifuged at 10,000× *g* for 1 h to remove larger vesicles and residual debris, followed by ultracentrifugation at 100,000× *g* for 90 min to collect the EVs-enriched pellet. The pellet, which contains the EVs-enriched fraction, was retained, and the supernatant was discarded. The pellet was washed once with PBS by resuspension and re-centrifugation at 100,000× *g* for 90 min, and the final pellet was resuspended in PBS [18].

To prepare MSCs^EGCG^-EVs, BMSCs were incubated with 5 μmol/L of EGCG for 24 h before EVs collection. After EGCG exposure, the cells were washed three times with PBS to remove residual EGCG and then cultured in serum-free medium for 48 h, after which conditioned medium was collected. EVs morphology was examined by transmission electron microscopy (TEM; Hitachi, Tokyo, Japan), and size distribution was measured by nanoparticle tracking analysis (NTA; NanoSight LM10, Malvern, UK). EVs and negative markers were evaluated by Western blotting as described below.

The total protein concentration of EVs was determined using a bicinchoninic acid (BCA) protein assay kit (Beyotime, Shanghai, China). Unless otherwise indicated, all treatments used 10 μg/mL of vesicle-associated protein. For the dose–response experiment, senescent HAECs with H_2_O_2_ exposure were treated with 0, 5, 10, and 20 μg/mL of MSCs^EGCG^-EVs [19]. Direct EGCG treatment of HAECs used 5 μmol/L for 24 h.

### 2.5. EVs Uptake Assay

EVs were labeled with DiI dye (Beyotime, China) according to the manufacturer’s instructions. After labeling, unbound DiI was removed by ultracentrifugation. HAECs were seeded in confocal dishes and incubated with DiI-labeled EVs at 10 μg/mL (protein concentration-based) for 6 h. Cells were washed with PBS, fixed with 4% paraformaldehyde (Servicebio, China), and counterstained with DAPI (Solarbio, China). Fluorescence was observed using a laser-scanning confocal microscope (Olympus, Tokyo, Japan) and quantified using ImageJ (version 1.8).

### 2.6. Cell Proliferation Assays

Cell proliferation was assessed using CCK-8 and EdU assays. For the CCK-8 assay, HAECs were seeded in 96-well plates and treated with 0, 5, 10, or 20 μg/mL of MSCs^EGCG^-EVs for 24 h. CCK-8 reagent (Beyotime, China) was added to each well and incubated for 2 h at 37 °C, and absorbance was measured at 450 nm. For the EdU assay, HAECs were incubated with 10 μmol/L of EdU (Solarbio, China) for 2 h and then fixed, permeabilized, and counterstained with DAPI. EdU-positive cells were visualized using a fluorescence microscope (Olympus, Japan) and quantified.

### 2.7. Senescence-Associated β-Galactosidase Staining

Cellular senescence was evaluated using a senescence-associated β-galactosidase staining kit (Beyotime, China). HAECs were washed with PBS, fixed with 4% paraformaldehyde for 15 min, and incubated with staining solution at 37 °C overnight. Blue-positive cells were observed under a light microscope (Olympus, Japan). The percentage of positive cells was calculated from at least three random fields per group.

### 2.8. Transwell Macrophage Migration Assay

Macrophage migration was assessed using Transwell chambers with 8 μm pores (Corning, Washington, DC, USA). HAECs were seeded in the lower chambers, exposed to H_2_O_2_ to induce senescence, and then treated with EGCG, MSCs-EVs, or MSCs^EGCG^-EVs as indicated. RAW264.7 macrophages were resuspended in serum-free medium and seeded into the upper chambers at 1 × 10^5^ cells/well. After 12 h of co-culture at 37 °C, non-migrated cells on the upper surface of the membrane were removed with a cotton swab. Cells that had migrated to the lower surface were fixed with 4% paraformaldehyde, stained with 0.1% crystal violet, and counted in five randomly selected fields per well using a light microscope.

### 2.9. Western Blotting

Cells or EVs were lysed in RIPA buffer containing protease and phosphatase inhibitors (Beyotime, China). Protein concentrations were determined using a BCA assay (Beyotime, China). Equal amounts of protein were separated by SDS-PAGE and transferred to PVDF membranes (Millipore, Darmstadt, Germany). Membranes were blocked with 5% non-fat milk for 1 h and incubated overnight at 4 °C with primary antibodies against iNOS (1:1000, 22226-1-AP, Proteintech, Wuhan, China), IL-6 (1:1000, 21865-1-AP, Proteintech), TNF-α (1:1000, 17590-1-AP, Proteintech), P16 (1:500, PA1210, Abmart, Shanghai, China), P21 (1:1000, PA9426, Abmart), BCL2 (1:1000, ab196495, Abcam, Cambridge, UK), BAX (1:1000, 50599-2, Proteintech), cleaved caspase-3 (1:500, 25128-1-AP, Proteintech), PINK1 (1:1000, 23274-1-AP, Proteintech), Parkin (1:1000, 2132, Cell Signaling Technology, Danvers, MA, USA), P62 (1:10,000, ab109012, Abcam), LC3B (1:2000, ab192890, Abcam), phospho-PI3K (Tyr607) (1:1000, ab182651, Abcam), PI3K (1:500, ab191606, Abcam), phospho-AKT (Ser473) (1:2000, 66444-1-Ig, Proteintech), AKT (1:5000, 60203-2-Ig, Proteintech), phospho-mTOR (Ser2448) (1:2000, 67778-1-Ig, Proteintech), mTOR (1:1000, ab134903, Abcam), phospho-NF-κB (Ser536) (1:2000, 80379-2-RR, Proteintech), NF-κB (1:1000, 66535-1-Ig, Proteintech), TSG101 (1:5000, 28283-1-AP, Proteintech), CD9 (1:500, bsm-52264R, Bioss), CD81 (1:1000, bsm-52640R, Bioss, Beijing, China), Calnexin (1:1000, bsm-52639R, Bioss), and β-actin (1:50,000, 66009-1-Ig, Proteintech). After washing, membranes were incubated with HRP-conjugated secondary antibodies for 1 h. Signals were visualized using enhanced chemiluminescence (ECL, Epizyme, Shanghai, China).

Gel and blot images were captured using Tanon 5200 at identical exposure settings. Brightness and contrast adjustments were applied uniformly to the entire image using ImageJ. No local modifications, splicing, or deletion of bands were performed. All original, uncropped images are provided in the Supplementary Materials for transparency.

### 2.10. JC-1 Staining for Mitochondrial Membrane Potential

Mitochondrial membrane potential (ΔΨm) was measured using a JC-1 staining kit (Beyotime, China). HAECs were incubated with JC-1 working solution at 37 °C for 20 min and observed using a fluorescence microscope. The red-to-green fluorescence intensity ratio was calculated using ImageJ.

### 2.11. Mitochondria–Lysosome Colocalization

HAECs were stained with MitoTracker Green (Beyotime, China) for 30 min and LysoTracker Red (Beyotime, China) for 25 min at 37 °C and then observed using a confocal microscope. Colocalization was quantified by calculating Pearson’s correlation coefficient using ImageJ.

### 2.12. Modulation of Mitochondrial Fission with Mdivi-1

HAECs were pretreated with 10 μmol/L of Mdivi-1 (MedChemExpress, Monmouth Junction, NJ, USA), a widely used mitochondrial fission modulator and putative Drp1 inhibitor, for 1 h before EV treatment. The cells were then co-treated with Mdivi-1 and MSCs^EGCG^-EVs for 24 h [20].

### 2.13. PINK1 Knockdown by siRNA

Small interfering RNAs targeting human PINK1 and a negative-control siRNA (si-NC) were designed and synthesized by Sangon Biotech. HAECs were transfected with 50 nM of siRNA using Lipofectamine 3000 (Thermo Fisher Scientific, Waltham, MA, USA) according to the manufacturer’s protocol. After 48 h, knockdown efficiency was evaluated by Western blotting, and the most efficient siRNA was used for subsequent H_2_O_2_ exposure experiments.

### 2.14. PI3K/AKT Pathway Activation

To activate PI3K/AKT signaling, HAECs were treated with 25 μg/mL of 740 Y-P (MedChemExpress, USA) for 1 h before the addition of MSCs^EGCG^-EVs, a cell-permeable phosphopeptide that binds the p85 regulatory subunit of PI3K and activates PI3K/AKT signaling. The cells were then co-treated with 740 Y-P and EVs for 24 h [21]. Pathway activation, mitophagy-associated protein expression, and senescence were evaluated.

### 2.15. Cellular Transmission Electron Microscopy

For cellular TEM, HAECs were harvested and fixed in 2.5% glutaraldehyde (Servicebio, China) at 4 °C overnight. After washing with PBS, cells were post-fixed in 1% osmium tetroxide for 1 h, dehydrated in a graded ethanol series, and embedded in epoxy resin. Ultrathin sections (60–80 nm) were cut, stained with uranyl acetate and lead citrate, and examined under a transmission electron microscope (Hitachi, Japan).

### 2.16. Statistical Analysis

All experiments were performed at least three independent times. Data are presented as mean ± standard deviation (SD). Statistical analyses were conducted using GraphPad Prism 12.0. Multiple-group comparisons were performed using a one-way analysis of variance followed by Bonferroni’s post hoc test. *p* < 0.05 was considered statistically significant.

## 3. Results

### 3.1. Characterization of EGCG-Primed BMSCs and BMSC-Derived EVs

**Figure 1:**
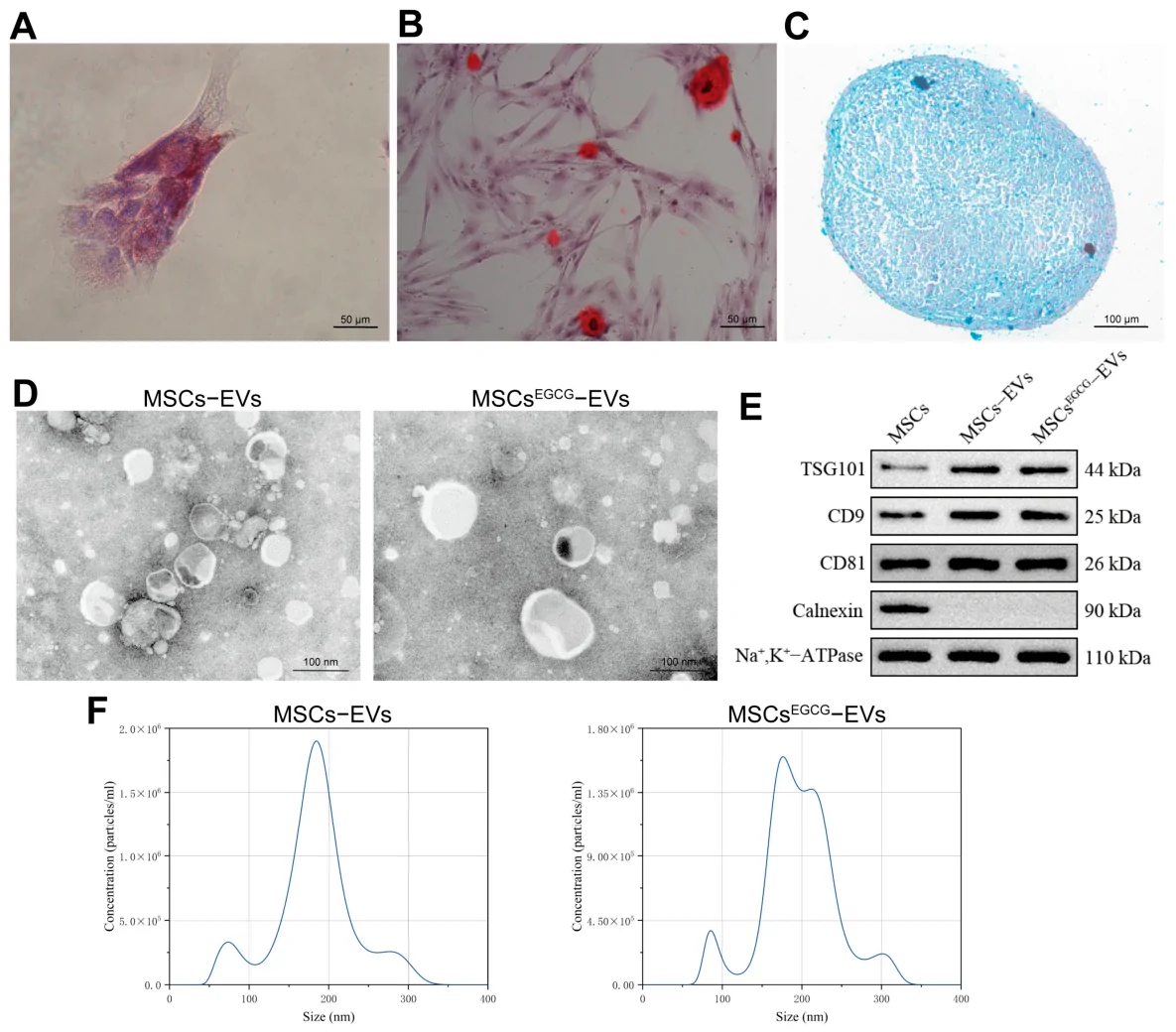
Characterization of BMSCs and BMSC-derived EVs. (**A**) Adipogenic differentiation assessed by Oil Red O staining (scale bar, 50 μm); (**B**) osteogenic differentiation assessed by Alizarin Red S staining (scale bar, 50 μm); (**C**) chondrogenic differentiation assessed by Alcian Blue staining (scale bar, 100 μm); (**D**) TEM images of MSCs-EVs and MSCs^EGCG^-EVs (scale bar, 100 nm); (**E**) Western blot analysis of TSG101, CD9, CD81, and Calnexin; and (**F**) NTA size distributions of MSCs-EVs and MSCs^EGCG^-EVs.

BMSCs underwent adipogenic, osteogenic, and chondrogenic differentiation (Figure 1A–C).

TEM showed that both MSCs-EVs and MSCs^EGCG^-EVs exhibited cup-shaped or spherical morphology (Figure 1D). TSG101, CD9, and CD81 were detected in both EV preparations, whereas the endoplasmic-reticulum marker Calnexin was not detected (Figure 1E). NTA indicated that most isolated particles were approximately 100–250 nm in diameter (Figure 1F).

### 3.2. MSCs^EGCG^-EVs Promote Proliferation of Senescent HAECs

**Figure 2:**
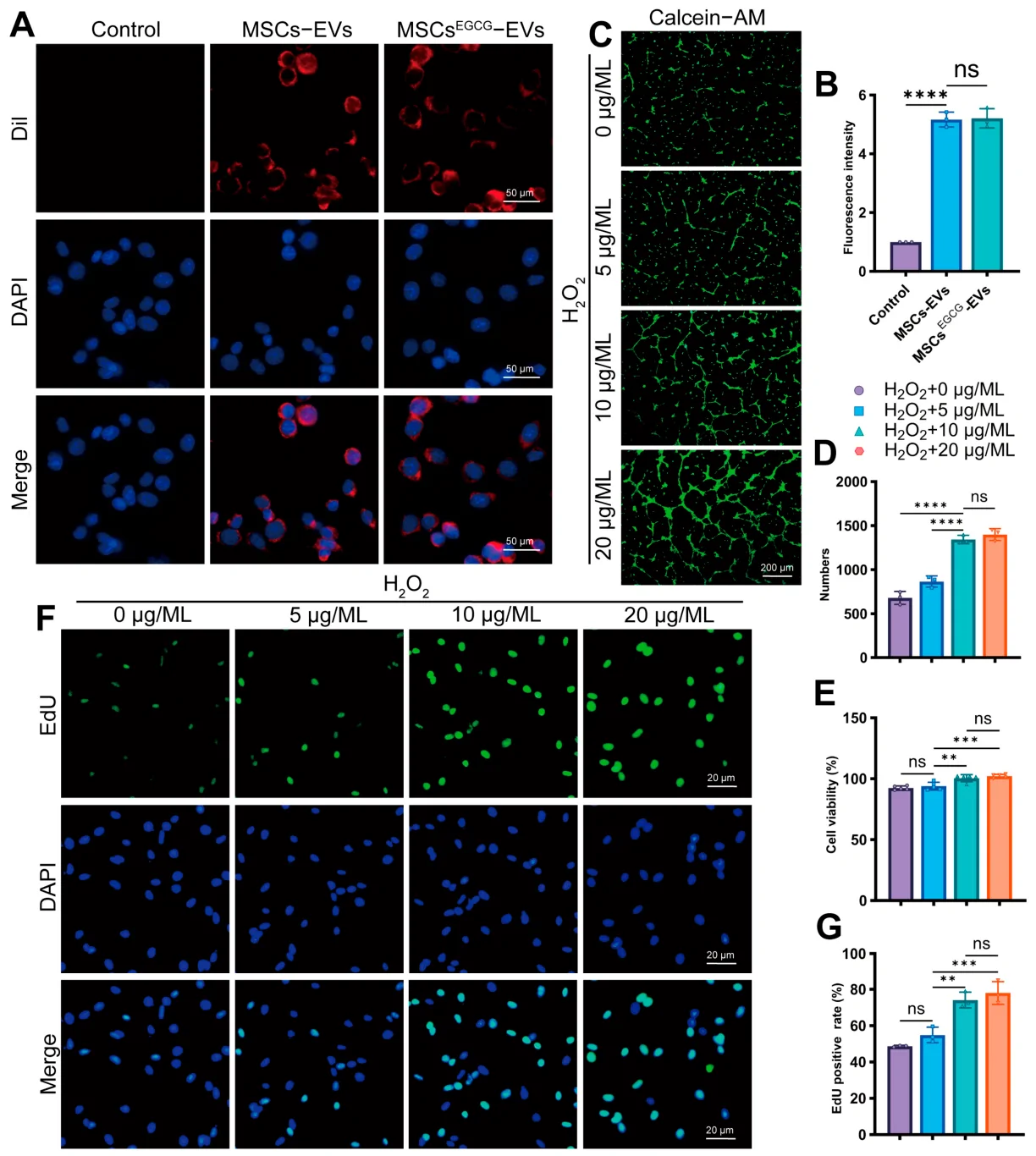
MSCs^EGCG^-EVs promote proliferation of senescent HAECs. (**A**) Internalization of DiI-labeled EVs (red) by HAECs; nuclei were counterstained with DAPI (blue; scale bar, 50 μm). (**B**) Fluorescence intensity quantification, *n* = 3. (**C**) HAECs were stained with Calcein-AM (green) to assess cell viability; scale bar, 50 μm. (**D**) Quantification of the number of Calcein-AM-positive cells per field, *n* = 3. (**E**) CCK-8 assay after treatment with the EV concentrations shown in the figure, *n* = 5. (**F**) EdU incorporation (green) with DAPI counterstaining (blue; scale bar, 20 μm). (**G**) Percentage of EdU-positive cells, *n* = 3. Data are presented as mean ± SD. ns, not significant; ** *p* < 0.01, *** *p* < 0.001, and **** *p* < 0.0001.

DiI-labeled EVs were detected in the cytoplasm of HAECs after co-incubation, whereas no DiI signal was detected in the control group (Figure 2A). Fluorescence intensity did not differ significantly between the MSCs-EV and MSCs^EGCG^-EV groups (Figure 2B).

In Calcein-AM live-cell fluorescence staining and CCK-8 assay, treatment with increasing concentrations (0, 5, 10, and 20 μg/mL) of MSCs^EGCG^-EVs increased the viability of senescent HAECs, with the largest changes observed at 10 and 20 μg/mL (Figure 2C–E). A concentration of 10 μg/mL was selected for subsequent experiments. EdU incorporation also increased after MSCs^EGCG^-EV treatment (Figure 2D,E).

### 3.3. MSCs^EGCG^-EVs Attenuate Senescence in HAECs and Reduce Macrophage Migration

**Figure 3:**
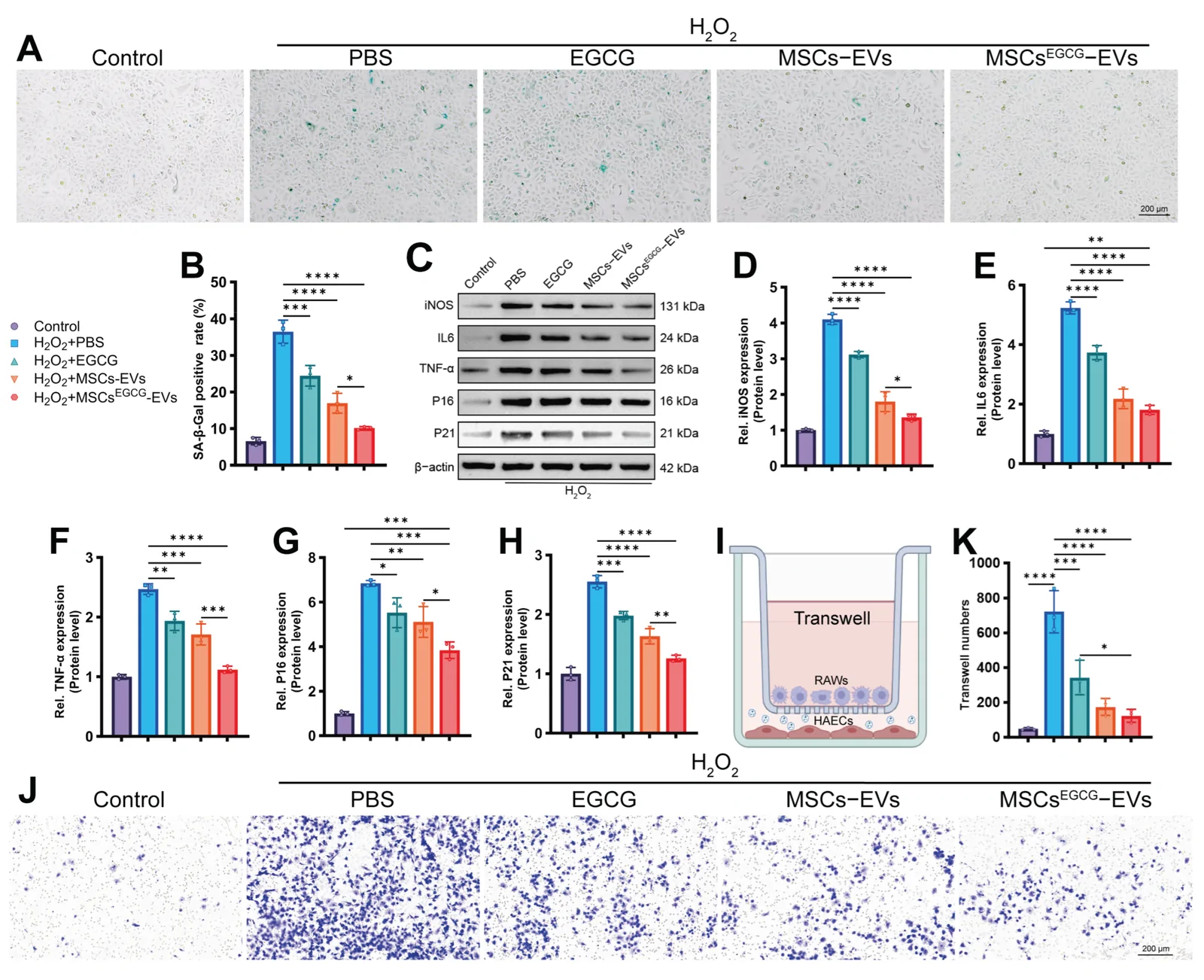
MSCs^EGCG^-EVs attenuate senescence in HAECs and reduce macrophage migration toward senescent HAECs. (**A**,**B**) Representative senescence-associated β-galactosidase staining and quantification of positive cells (scale bar, 200 μm), *n* = 3. (**C**–**H**) Western blot analysis and quantification of iNOS, IL-6, TNF-α, P16, and P21, *n* = 3. (**I**) Schematic of the Transwell co-culture assay. (**J**,**K**) Representative crystal-violet-stained RAW264.7 macrophages and quantification of migrated cells (scale bar, 200 μm), *n* = 3. Data are presented as mean ± SD. * *p* < 0.05, ** *p* < 0.01, *** *p* < 0.001, and **** *p* < 0.0001.

H_2_O_2_-induced senescence was evaluated by senescence-associated β-galactosidase staining. The percentage of positive cells was higher in the PBS-treated model group than in the untreated control group. EGCG or MSCs-EVs partially reduced the percentage of positive cells, whereas MSCs^EGCG^-EVs produced the largest reduction (Figure 3A,B).

H_2_O_2_ also increased the expression of iNOS, IL-6, TNF-α, P16, and P21. These increases were attenuated to different extents by EGCG, MSCs-EVs, and MSCs^EGCG^-EVs, with the strongest reduction in the MSCs^EGCG^-EV group (Figure 3C–H).

In the Transwell assay, RAW264.7 macrophage migration toward H_2_O_2_-senescent HAECs was greater than migration toward untreated control HAECs. Treatment of senescent HAECs with EGCG or MSCs-EVs reduced macrophage migration, whereas MSCs^EGCG^-EVs produced the largest reduction (Figure 3I–K).

### 3.4. MSCs^EGCG^-EVs Improve Mitochondrial Morphology and Enhance Mitochondrial Quality Control-Associated Changes in Senescent HAECs

**Figure 4:**
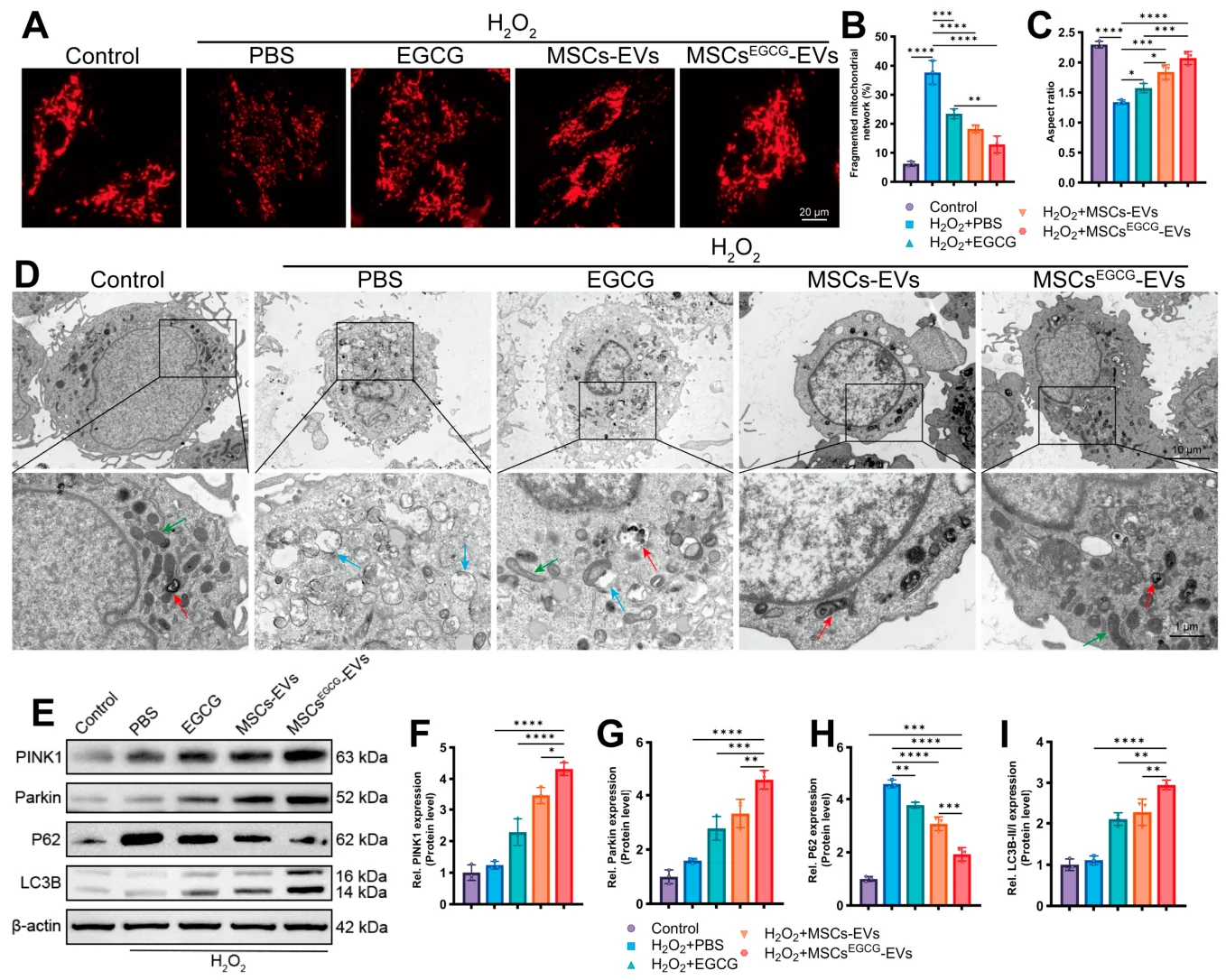
MSCs^EGCG^-EVs improve mitochondrial morphology and enhance mitochondrial quality control-associated changes in senescent HAECs. (**A**) MitoTracker imaging of mitochondrial networks (scale bar, 20 μm). (**B**,**C**) Percentage of fragmented mitochondrial networks and mitochondrial aspect ratio, *n* = 3. (**D**) TEM images of mitochondrial morphology and autophagic structures; green arrows indicate morphologically preserved mitochondria, blue arrows indicate damaged mitochondria, and red arrows indicate mitophagy-associated structures; this ultrastructural analysis suggests, but does not directly quantify, mitophagic flux. (scale bars, 10 μm and 1 μm). (**E**–**I**) Western blot analysis and quantification of PINK1, Parkin, P62, and LC3B, *n* = 3. Data are presented as mean ± SD. * *p* < 0.05, ** *p* < 0.01, *** *p* < 0.001, and **** *p* < 0.0001.

MitoTracker imaging showed an interconnected mitochondrial network in untreated HAECs, whereas H_2_O_2_-treated cells displayed marked mitochondrial fragmentation, a greater percentage of fragmented mitochondrial networks, and a lower aspect ratio. EGCG, MSCs-EVs, and MSCs^EGCG^-EVs improved these morphological measures to different extents, with the largest changes in the MSCs^EGCG^-EV group (Figure 4A–C).

TEM showed preserved mitochondrial morphology in untreated control cells and swollen, damaged mitochondria after H_2_O_2_ exposure. MSCs^EGCG^-EV treatment increased autophagic structures associated with mitochondria (Figure 4D). These data are consistent with enhanced mitophagy-associated activity, but they do not directly demonstrate mitophagic flux.

H_2_O_2_ modestly increased PINK1 and Parkin expression relative to the untreated control. MSCs^EGCG^-EVs further increased PINK1 and Parkin, reduced P62, and increased the LC3B-II/I ratio; these changes were less pronounced after EGCG or MSCs-EV treatment (Figure 4E–I).

### 3.5. MSCs^EGCG^-EVs Restore Mitochondrial Membrane Potential and Increase Mitochondria–Lysosome Colocalization

**Figure 5:**
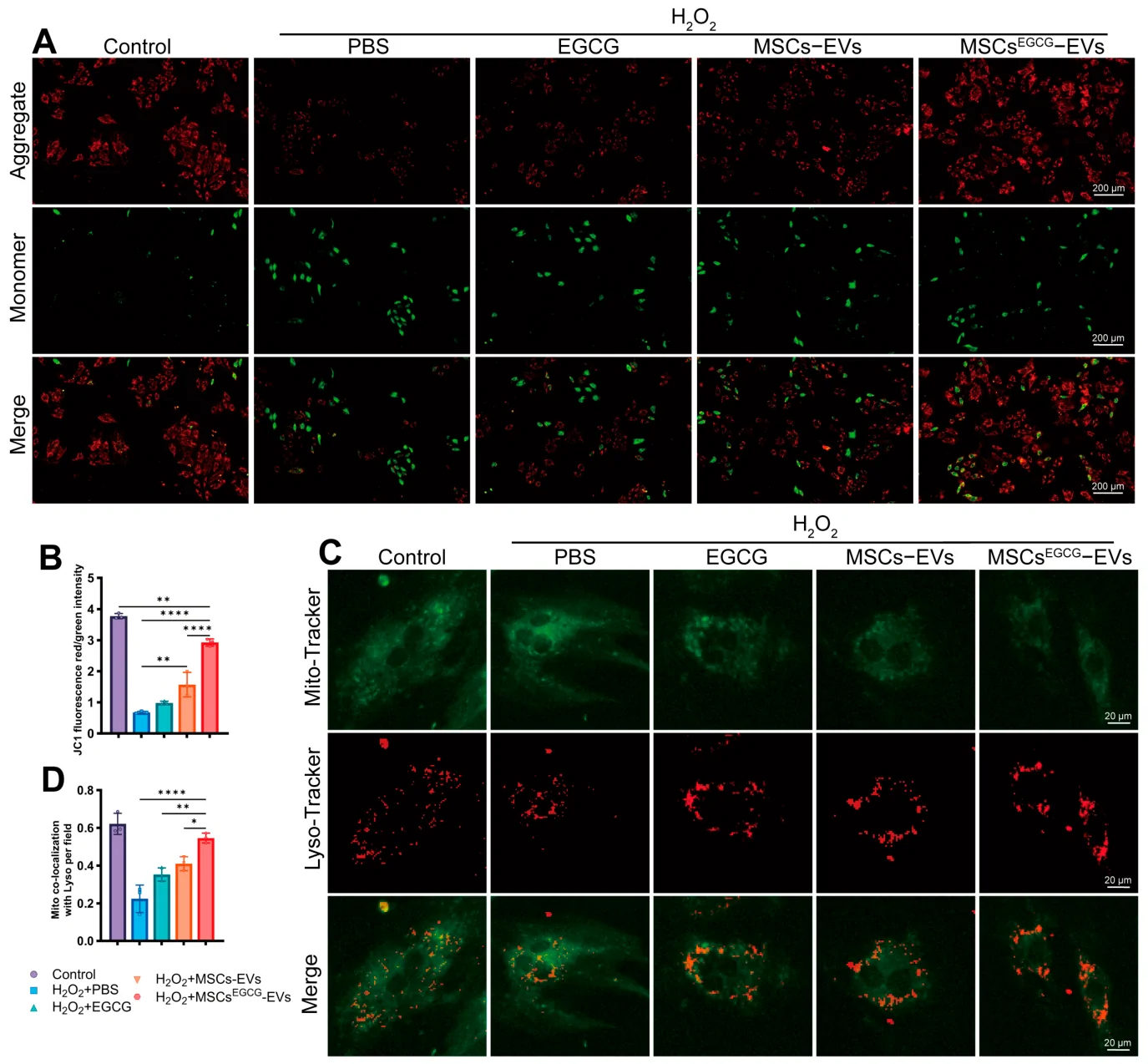
MSCs^EGCG^-EVs restore mitochondrial membrane potential and increase mitochondria–lysosome colocalization. (**A**) JC-1 staining of HAECs (scale bar, 200 μm). (**B**) Quantification of the red-to-green fluorescence intensity ratio, *n* = 3. (**C**) MitoTracker (green) and LysoTracker (red) staining; yellow signals in merged images indicate colocalization (scale bar, 20 μm). (**D**) Quantification of mitochondria–lysosome colocalization, which is consistent with increased delivery of mitochondria to lysosomes but is not a direct measurement of mitophagic flux, *n* = 3. Data are presented as mean ± SD. * *p* < 0.05, ** *p* < 0.01 and **** *p* < 0.0001.

Untreated control cells showed predominantly red JC-1 fluorescence, consistent with preserved ΔΨm, whereas H_2_O_2_ shifted the signal toward green fluorescence. MSCs^EGCG^-EVs produced the largest restoration of the red-to-green fluorescence ratio; EGCG and MSCs-EVs produced intermediate changes (Figure 5A,B).

MSCs^EGCG^-EVs also increased the overlap between MitoTracker and LysoTracker signals (Figure 5C,D), consistent with the increased delivery of mitochondria to lysosomal compartments.

### 3.6. Mdivi-1 Attenuates the Protective Effects of MSCs^EGCG^-EVs

**Figure 6:**
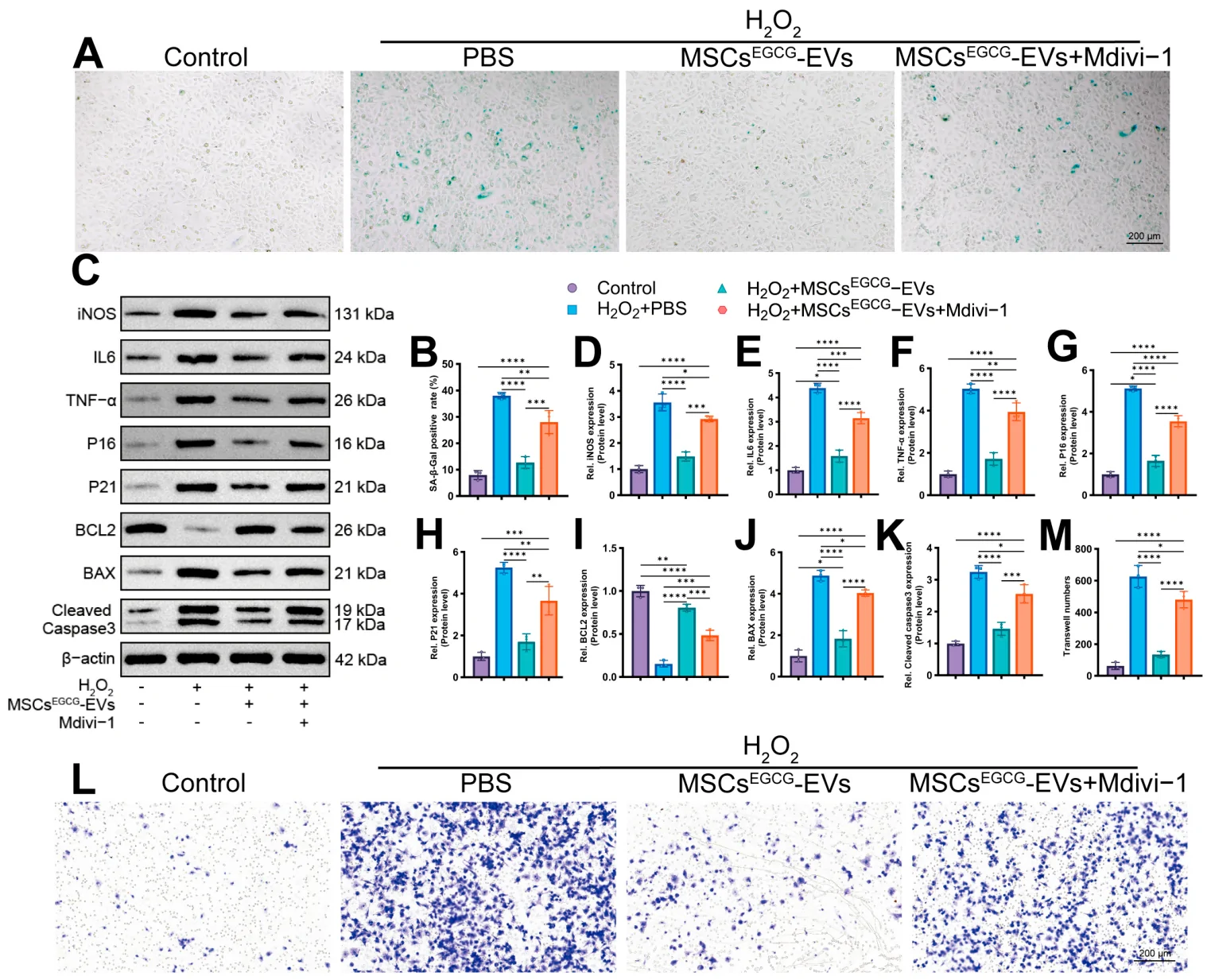
Mdivi-1 attenuates the protective effects of MSCs^EGCG^-EVs. (**A**,**B**) Representative senescence-associated β-galactosidase staining and quantification of positive cells (scale bar, 200 μm), *n* = 3. (**C**–**K**) Western blot analysis and quantification of iNOS, IL-6, TNF-α, P16, P21, BCL2, BAX, and cleaved caspase-3. (**L**,**M**) Representative Transwell migration images and quantification of migrated RAW264.7 macrophages (scale bar, 200 μm), *n* = 3. Data are presented as mean ± SD. * *p* < 0.05, ** *p* < 0.01, *** *p* < 0.001, and **** *p* < 0.0001.

To examine whether mitochondrial dynamics and mitochondrial quality control-associated processes contribute to the effects of MSCs^EGCG^-EVs, HAECs were co-treated with Mdivi-1. Mdivi-1 alone does not induce senescence but exacerbates H_2_O_2_-induced senescence (Supplementary Figure S1A–C). MSCs^EGCG^-EVs reduced H_2_O_2_-induced senescence-associated β-galactosidase positivity, whereas Mdivi-1 attenuated this effect (Figure 6A,B).

MSCs^EGCG^-EVs reduced iNOS, IL-6, TNF-α, P16, P21, BAX, and cleaved caspase-3 and increased BCL2. Co-treatment with Mdivi-1 attenuated these protein expression changes (Figure 6C–K).

Mdivi-1 also attenuated the MSCs^EGCG^-EV-associated reduction in RAW264.7 macrophage migration toward senescent HAECs (Figure 6L,M).

### 3.7. Effects of PINK1 Knockdown in the Presence of MSCsEGCG-EVs

Among the tested siRNAs, si-PINK1-B produced the greatest reduction in PINK1 protein expression and was selected for subsequent experiments (Supplementary Figure S2A,B).

**Figure 7:**
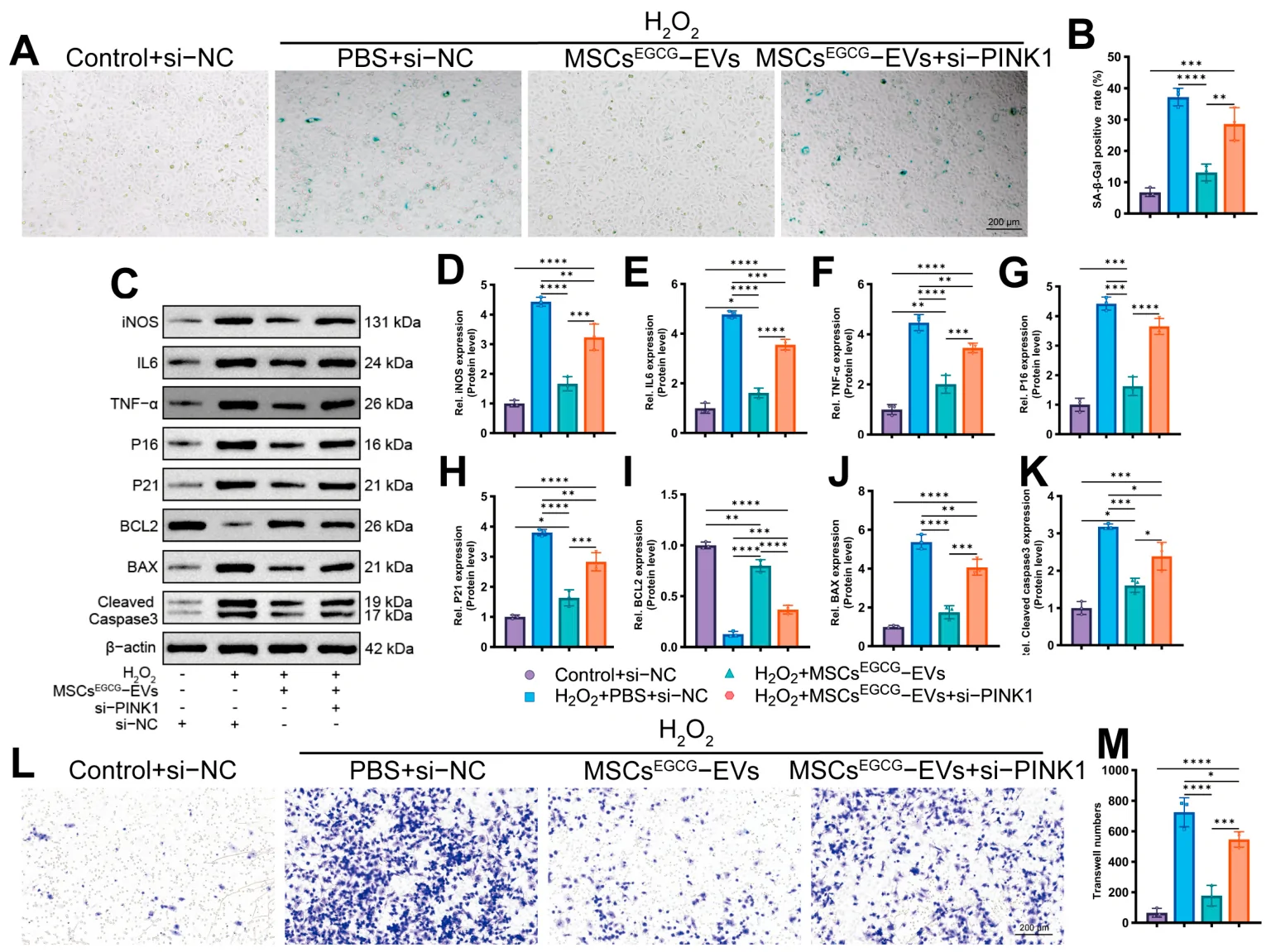
Effects of PINK1 knockdown in the presence of MSCsEGCG-EVs. (**A**,**B**) Representative senescence-associated β-galactosidase staining and quantification of positive cells (scale bar, 200 μm), *n* = 3; (**C**–**K**) Western blot analysis and quantification of iNOS, IL-6, TNF-α, P16, P21, BCL2, BAX, and cleaved caspase-3, *n* = 3. (**L**,**M**) Representative Transwell migration images and quantification of migrated RAW264.7 macrophages (scale bar, 200 μm), *n* = 3. Data are presented as mean ± SD. * *p* < 0.05, ** *p* < 0.01, *** *p* < 0.001, and **** *p* < 0.0001.

PINK1 knockdown alone did not alter P16 or P21 levels, but it intensified H_2_O_2_-induced senescence (Supplementary Figure S1D–F). Furthermore, PINK1 knockdown attenuated the reduction in senescence-associated β-galactosidase positivity produced by MSCs^EGCG^-EVs (Figure 7A,B).

In the presence of PINK1 knockdown, the MSCsEGCG-EV-associated reductions in iNOS, IL-6, TNF-α, P16, P21, BAX, and cleaved caspase-3 and the increase in BCL2 were less pronounced(Figure 7C–K). Fluorescence colocalization analysis showed that PINK1 knockdown attenuated EV-induced mitochondria–lysosome colocalization, supporting a role for PINK1 in mitochondrial quality control in this model (Supplementary Figure S2C,D).

PINK1 knockdown likewise attenuated the MSCs^EGCG^-EV-associated reduction in RAW264.7 macrophage migration toward senescent HAECs (Figure 7L,M).

### 3.8. PI3K Activation Attenuates the Effects of MSCs^EGCG^-EVs on Mitochondrial Quality Control-Associated Proteins, Senescence, and Macrophage Migration

**Figure 8:**
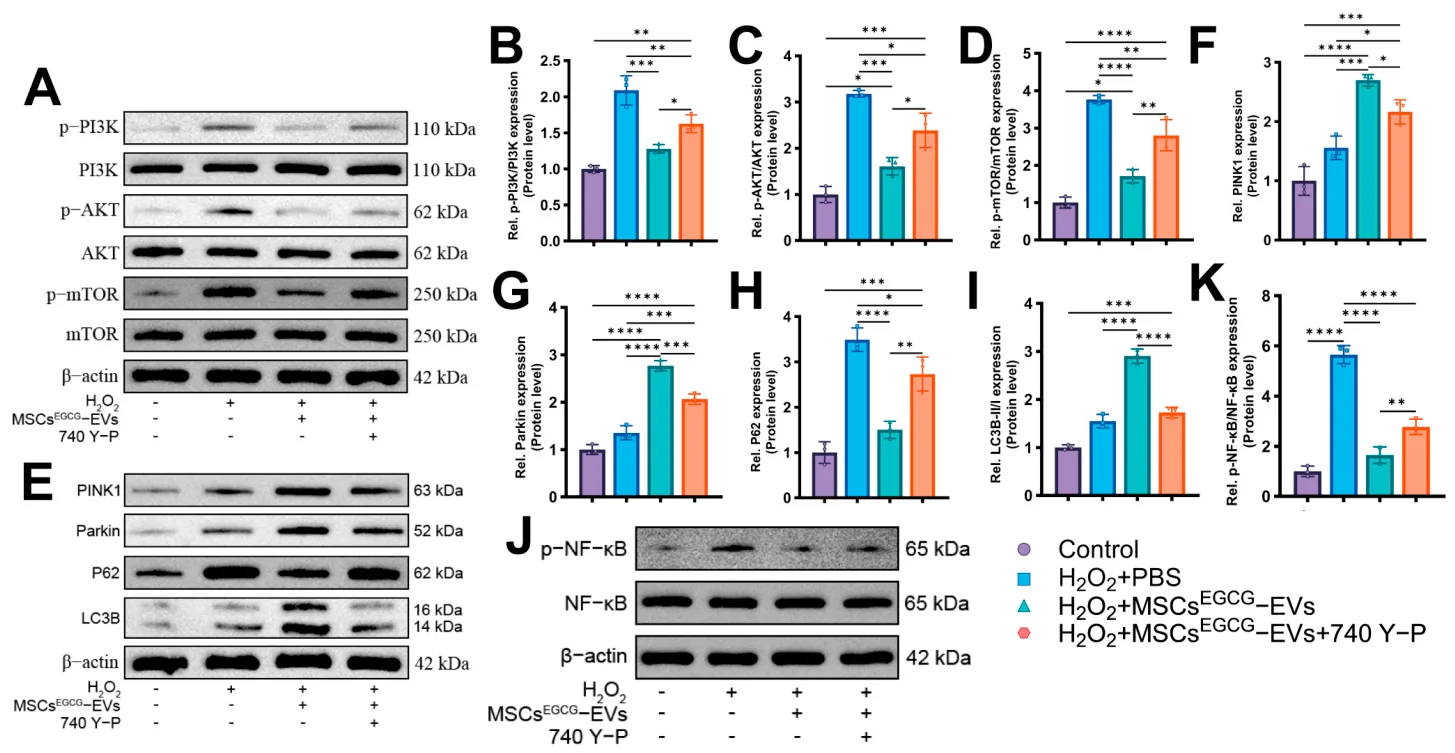
PI3K activation attenuates the effects of MSCs^EGCG^-EVs on mitochondrial quality control-associated proteins, senescence, and macrophage migration. (**A**–**D**) Western blot analysis and quantification of phospho-PI3K (Tyr607)/PI3K, phospho-AKT (Ser473)/AKT, and phospho-mTOR (Ser2448)/mTOR, *n* = 3. (**E**–**I**) Western blot analysis and quantification of PINK1, Parkin, P62, and LC3B, *n* = 3. (**J**,**K**) Western blot analysis and quantification of phospho-NF-κB p65 (Ser536)/NF-κB p65, *n* = 3. Data are presented as mean ± SD. * *p* < 0.05, ** *p* < 0.01, *** *p* < 0.001, and **** *p* < 0.0001.

H_2_O_2_ increased phosphorylation of PI3K, AKT, and mTOR relative to the untreated control. MSCs^EGCG^-EVs reduced phosphorylation of these proteins, whereas the PI3K activator 740 Y-P attenuated these reductions (Figure 8A–D). 740 Y-P treatment alone had no effect on senescence markers, whereas it further aggravated H_2_O_2_-induced upregulation of P16 and P21 (Supplementary Figure S1G–I).

In addition, 740 Y-P attenuated the MSCs^EGCG^-EV-associated increases in PINK1, Parkin, and the LC3B-II/I ratio; increased P62 relative to MSCs^EGCG^-EV treatment alone (Figure 8E–K); and was associated with a less pronounced reduction in senescence-associated β-galactosidase positivity (Supplementary Figure S3A,B). Western blotting further showed that H_2_O_2_ increased the phosphorylation of NF-κB p65 (Ser536) relative to the untreated control. MSCs^EGCG^-EVs reduced p65 phosphorylation, whereas this reduction was less pronounced in the presence of 740 Y-P (Figure 8J,K). Total p65 levels did not differ significantly among the groups.

Activation of PI3K/AKT signaling by 740 Y-P also attenuated the MSCs^EGCG^-EV-associated reduction in RAW264.7 macrophage migration toward senescent HAECs (Supplementary Figure S3C,D).

## 4. Discussion

Endothelial dysfunction, oxidative stress, and chronic inflammation are central to AS, and endothelial cell senescence can contribute to disease initiation and progression [22,23]. Mitochondrial damage, excessive reactive oxygen species accumulation, and impaired mitophagy are also implicated in endothelial senescence [6]. In the present in vitro study, EVs from EGCG-primed BMSCs reduced multiple features of H_2_O_2_-induced HAEC senescence while increasing PINK1/Parkin- and LC3B-associated changes and reducing PI3K/AKT/mTOR activation.

MSC-derived EVs have been investigated as paracrine mediators and delivery vehicles in regenerative medicine [10]. Compared with cell transplantation, an EVs-based approach may prevent some risks associated with cell survival, immune rejection, and uncontrolled cell behavior [24]. However, the activity of unprimed MSCs-EVs may be limited under oxidative and inflammatory conditions, motivating the investigation of priming strategies [23]. In this study, EGCG priming did not produce an evident change in EVs morphology or marker profiles, while MSCs^EGCG^-EVs generally produced larger functional changes than unprimed MSCs-EVs. The active cargo responsible for this difference was not identified.

Cellular senescence is characterized by persistent stress responses, cell-cycle arrest, and SASP activation, which can amplify local inflammation and vascular aging [25]. H_2_O_2_ increased senescence-associated β-galactosidase positivity and the abundance of iNOS, IL-6, TNF-α, P16, and P21. These changes were attenuated most strongly by MSCs^EGCG^-EVs, consistent with the anti-senescent and anti-inflammatory effects observed in the HAEC model. Senescent HAECs also promoted RAW264.7 macrophage migration in the Transwell system, and this response was reduced by MSCs^EGCG^-EVs. The migration results extend the cellular phenotype to a functional inflammatory readout, although the specific HAEC-derived chemotactic mediators were not identified.

Mitochondrial dysfunction and impaired mitophagy have important roles in endothelial senescence and AS [26]. Damaged mitochondria can increase oxidative stress, disrupt membrane potential, and activate apoptotic signaling [27], whereas inadequate mitophagy permits dysfunctional mitochondria to accumulate [28]. MSCs^EGCG^-EVs improved the JC-1 red-to-green ratio, increased PINK1 and Parkin, increased the LC3B-II/I ratio, reduced P62, and increased mitochondria–lysosome colocalization. Together with the TEM findings, these observations support enhanced mitophagy-associated activity [29]. Although mitochondria–lysosome colocalization was increased and PINK1 knockdown attenuated this response, this assay does not directly measure mitophagic flux. Therefore, the current data support PINK1/Parkin-associated mitochondrial quality control rather than definitive PINK1/Parkin-mediated mitophagy. Future studies using lysosomal inhibitors, mKeima or mito-QC reporters, and Parkin mitochondrial translocation are required.

The involvement of this pathway was further examined using Mdivi-1 and PINK1 siRNA [30,31]. Mdivi-1 attenuated the anti-senescent, apoptosis-associated protein, and macrophage migration changes produced by MSCs^EGCG^-EVs. Because pharmacological modulators may have off-target effects, this result should not be interpreted as specific proof of mitochondrial quality control inhibition. The attenuation observed in the presence of PINK1 knockdown supports an association between PINK1-related mitochondrial quality control and the observed endothelial and macrophage migration phenotypes but does not establish exclusive PINK1 dependence. This interpretation is consistent with previous work linking PINK1/Parkin signaling to vascular protection [29].

PI3K/AKT/mTOR signaling regulates growth, metabolism, autophagy, and cellular senescence [15,16]. Excessive pathway activation can suppress autophagic processes and aggravate mitochondrial dysfunction [32]. MSCs^EGCG^-EVs reduced H_2_O_2_-induced phosphorylation of PI3K, AKT, and mTOR. The PI3K activator 740 Y-P attenuated these signaling changes, reduced the mitochondrial quality control-associated protein response, partially restored senescence-associated β-galactosidase positivity, and increased macrophage migration toward senescent HAECs relative to MSCs^EGCG^-EV treatment alone. Furthermore, by inhibiting the PI3K/AKT/mTOR pathway, MSCs^EGCG^-EVs reduce AKT-mediated phosphorylation and activation of NF-κB, thereby inhibiting p65 nuclear translocation and the transcriptional initiation of inflammatory genes such as iNOS, IL-6, and TNF-α, while PINK1/Parkin-associated mitochondrial quality control clears damaged mitochondria [33]. Collectively, these findings identify NF-κB p65 as a critical downstream effector through which PI3K/AKT/mTOR signaling promotes endothelial inflammation and senescence and indicate that MSCs^EGCG^-EVs suppress this inflammatory axis. These data are consistent with functional coupling among PI3K/AKT/mTOR signaling, PINK1/Parkin-associated mitochondrial quality control, endothelial senescence, and the endothelial inflammatory phenotype in this model. What is noteworthy is that the intervention-only controls showed that Mdivi-1, PINK1 knockdown, and 740 Y-P alone aggravated H_2_O_2_-induced senescence. Therefore, the EV experiments were interpreted relative to the corresponding H_2_O_2_ + intervention controls rather than to H_2_O_2_ alone. This distinction is important because these interventions have independent effects in the H_2_O_2_ model.

This study has several limitations. First, all functional experiments used an H_2_O_2_-induced in vitro model; no atherosclerotic lesion or in vivo therapeutic effect was evaluated. Validation in ApoE−/− or LDLR−/− models is therefore required [34,35]. Second, rat BMSC-derived EVs were tested in human HAECs, and murine RAW264.7 macrophages were used in the migration assay, creating cross-species systems that should be confirmed using human MSCs and human macrophages. Third, the miRNAs, proteins, or lipids responsible for the enhanced activity of MSCs^EGCG^-EVs remain unknown; comparative cargo profiling may identify relevant mediators [36]. The Transwell assay likewise did not identify the individual chemotactic factors responsible for macrophage migration. Fourth, the study did not use lysosomal blockade or a dedicated reporter to quantify mitophagic flux. Finally, long-term safety, biodistribution, and pharmacokinetics require evaluation before clinical translation.

## 5. Conclusions

EVs derived from EGCG-primed BMSCs attenuated H_2_O_2_-induced endothelial senescence, as shown by increased proliferation and a reduction in β-Gal^+^ cells from 36.47% to 10.17%, and decreased RAW264.7 macrophage migration from 720.33 to 123.67. These EVs restored mitochondrial membrane potential, enhanced PINK1/Parkin-associated mitochondrial quality control markers and suppressed PI3K/AKT/mTOR phosphorylation. Relative to the corresponding H_2_O_2_ + intervention controls, MSCsEGCG-EVs retained partial protective effects in the presence of Mdivi-1, PINK1 knockdown, and the PI3K activator 740 Y-P, although the magnitude of protection was smaller than that observed without these interventions. These findings suggest that mitochondrial dynamics, PINK1-associated mitochondrial quality control, and PI3K/AKT/mTOR signaling contribute to the EV-mediated protection and are consistent with a model in which inhibition of PI3K/AKT/mTOR may disinhibit PINK1/Parkin-associated mitochondrial quality control, thereby alleviating endothelial senescence and SASP-driven macrophage recruitment, although additional mechanisms cannot be excluded. However, because the evidence derives from cross-species in vitro models and no atherosclerotic lesion model was tested, further in vivo and human cell studies are required before therapeutic conclusions can be drawn. These findings suggest that EGCG-primed BMSC-derived EVs represent a potential cell-free strategy for targeting endothelial senescence, but their efficacy and safety in atherosclerosis remain to be established.

## Data Availability

The data presented in this study are available from the corresponding author upon reasonable request.

## Supplementary Materials

The following supporting information can be downloaded at: https://www.mdpi.com/article/10.3390/life16101737/s1. Supplementary Table S1. Sequences of siRNAs used in this study; Supplementary Figure S1. (A–C) Representative Western blots and quantification of P16 and P21 in HAECs treated with Mdivi-1. (D–F) Representative Western blots and quantification of P16 and P21 in HAECs treated with si-PINK1. (G–I) Representative Western blots and quantification of P16 and P21 in HAECs treated with 740 Y-P; Supplementary Figure S2. (A,B) Western blot validation of PINK1 knockdown, *n* = 3. (C) MitoTracker (green) and LysoTracker (red) staining; yellow signals in merged images indicate colocalization (scale bar, 20 μm). (D) Quantification of mitochondria–lysosome colocalization, *n* = 3. Data are presented as mean ± SD. **** *p* < 0.0001; Supplementary Figure S3. (A,B) Representative senescence-associated β-galactosidase staining and quantification of positive cells (scale bar, 200 μm), *n* = 3. (C,D) Representative Transwell migration images and quantification of migrated RAW264.7 macrophages (scale bar, 200 μm), *n* = 3. Data are presented as mean ± SD. ** *p* < 0.01, *** *p* < 0.001, and **** *p* < 0.0001.

## Abbreviations

The following abbreviations are used in this manuscript:

AS	Atherosclerosis
BMSC	Bone marrow mesenchymal stem cell
CCK-8	Cell Counting Kit-8
EGCG	Epigallocatechin gallate
HAEC	Human aortic endothelial cell
MSC-EVs	Extracellular vesicles derived from unprimed mesenchymal stem cells
MSCsEGCG-EVs	Extracellular vesicles derived from EGCG-primed mesenchymal stem cells
NTA	Nanoparticle tracking analysis
ROS	Reactive oxygen species
SASP	Senescence-associated secretory phenotype
TEM	Transmission electron microscopy
ΔΨm	Mitochondrial membrane potential
IACUC	Institutional Animal Care and Use Committee
SPF	Specific-pathogen-free
