# Supplementary material for: Epigallocatechin Gallate-Primed Bone Marrow Mesenchymal Stem Cell-Derived Extracellular Vesicles Attenuate Endothelial Senescence by Modulating PI3K/AKT/mTOR Signaling and PINK1-Associated Mitochondrial Quality Control
Source: Life (Basel). 2026 Oct 16;16(10):1737. doi: 10.3390/life16101737 (PMC13706218; doi:10.3390/life16101737)
Supplement: Supplementary file 1 [file life-16-01737-s001.zip › Supplementary files.pdf]

Epigallocatechin Gallate-Primed Bone Marrow Mesenchymal Stem Cell-Derived Extracellular Vesicles Attenuate Endothelial Senescence by Modulating PI3K/AKT/mTOR Signaling and PINK1-Associated Mitochondrial Quality Control

Yifan Tao, Yangli He, Tian Xie and Min Zeng*

Author information
Affiliations
Institute of Cardiovascular Diseases, Hainan Medical University, Haikou, 571199, China
*** Correspondence:**
Min Zeng
email: zengmin@muhn.edu.cn
Institute of Cardiovascular Diseases, Hainan Medical University, Haikou, 571199, China

Supplementary Materials

Supplementary Table S1. Sequences of siRNAs used in this study.

Name	Relative sequences (5’-3’)
si-NC Sense	UUCUCCGAACGUGUCACGUTT
si-PINK1-A Sense	CAGACAUCUGAAAAGUGAA
si-PINK1-B Sense	GCAAAUGUGCUUCAUCUAA
si-PINK1-C Sense	GACGCUGUUCCUCGUUAUGAA

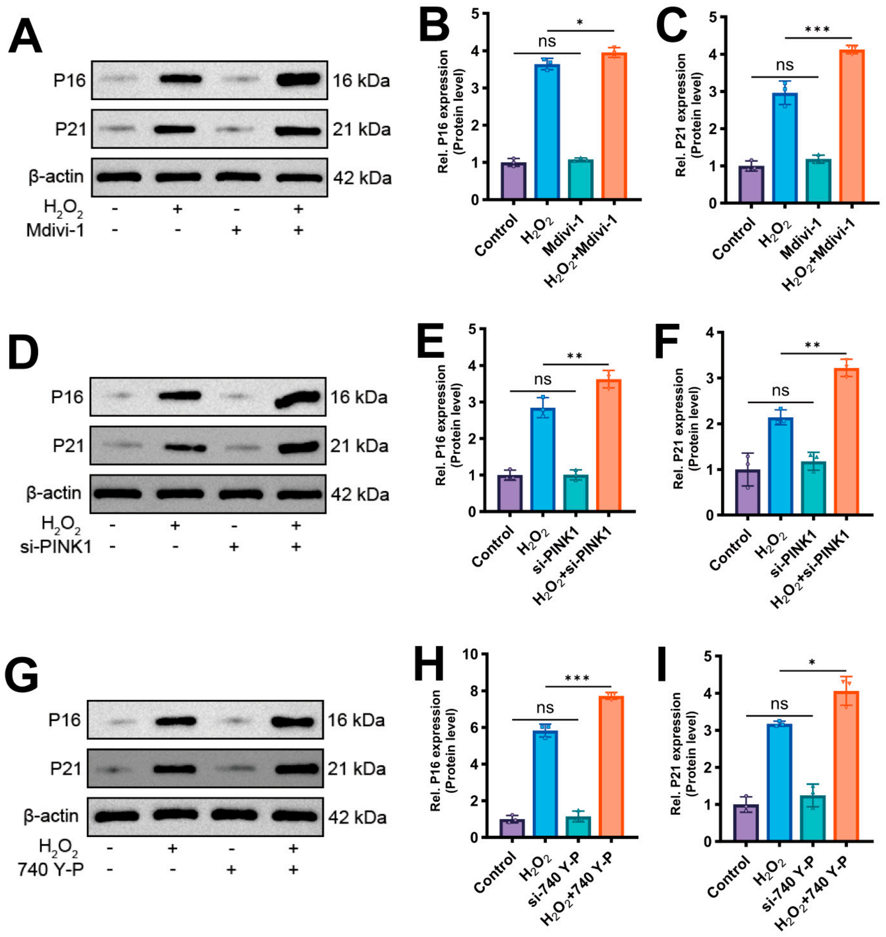

Supplementary Figure S1. (A-C) Representative Western blots and quantification of P16 and P21 in HAECs treated with Mdivi-1. (D-F) Representative Western blots and quantification of P16 and P21 in HAECs treated with si-PINK1. (G-I) Representative Western blots and quantification of P16 and P21 in HAECs treated with 740 Y-P.

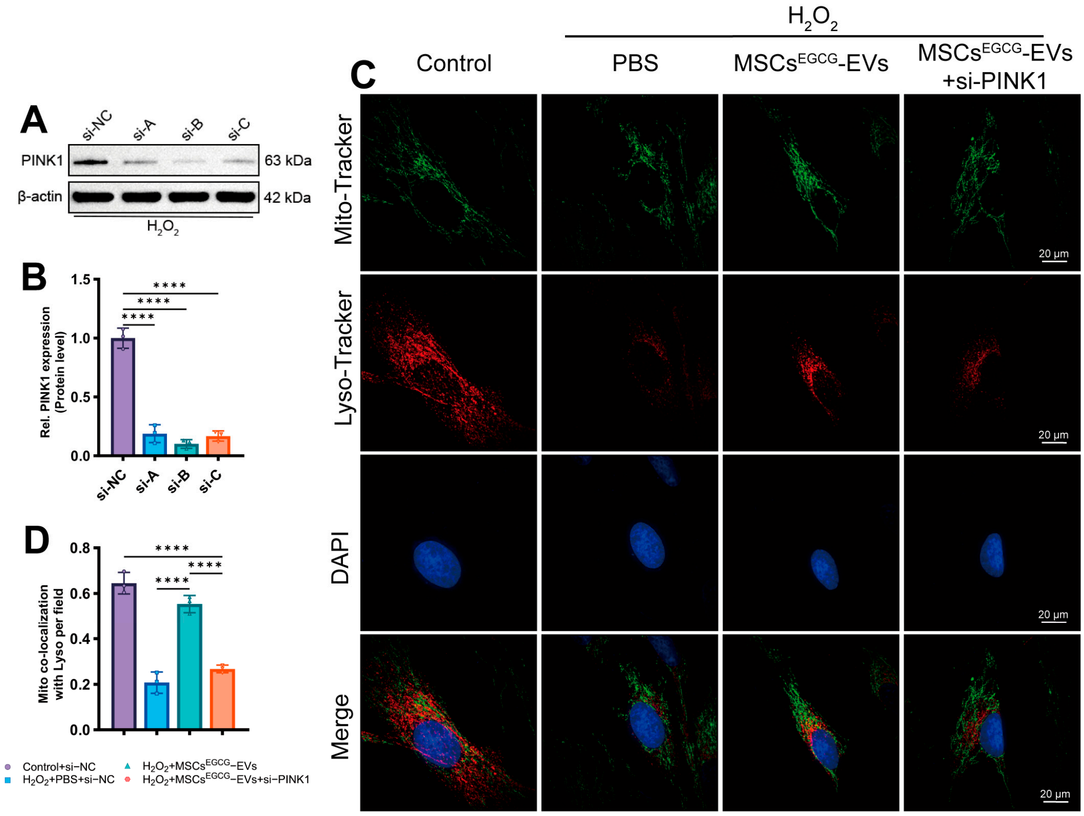

Supplementary Figure S2. (A,B) Western blot validation of PINK1 knockdown, n=3. (C) MitoTracker (green) and LysoTracker (red) staining; yellow signals in merged images indicate colocalization (scale bar, 20 μm). (D) Quantification of mitochondria–lysosome colocalization, n=3. Data are presented as mean ± SD. ****p < 0.0001.

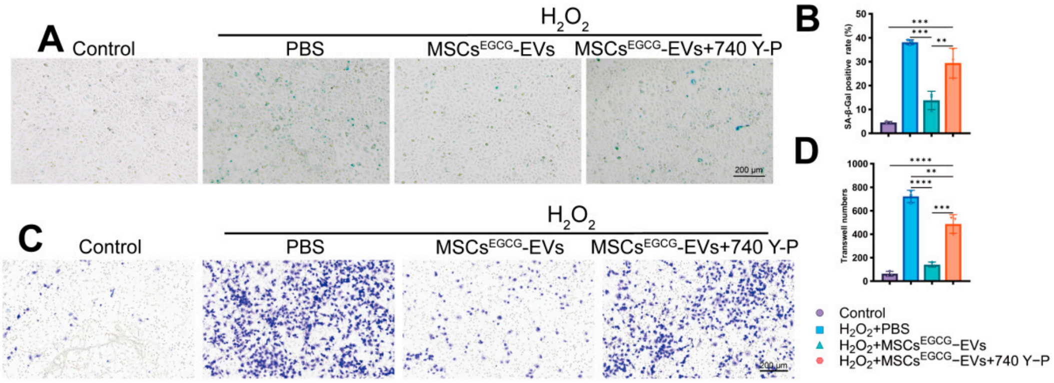

Supplementary Figure S3. (A,B) Representative senescence-associated β-galactosidase staining and quantification of positive cells (scale bar, 200 μm), n=3. (C,D) Representative Transwell migration images and quantification of migrated RAW264.7 macrophages (scale bar, 200 μm), n=3. Data are presented as mean ± SD. **p < 0.01, ***p < 0.001, and ****p < 0.0001.
